# Identification of Low Molecular Weight Proteins and Peptides from *Schistosoma mekongi* Worm, Egg and Infected Mouse Sera

**DOI:** 10.3390/biom11040559

**Published:** 2021-04-11

**Authors:** Tipparat Thiangtrongjit, Nattapon Simanon, Poom Adisakwattana, Yanin Limpanont, Phiraphol Chusongsang, Yupa Chusongsang, Onrapak Reamtong

**Affiliations:** 1 Department of Molecular Tropical Medicine and Genetics, Faculty of Tropical Medicine, Mahidol University, Bangkok 10400, Thailand; tipparat.thi@mahidol.ac.th; 2 National Omics Center (NOC), National Science and Technology Development Agency, Pathum Thani 12120, Thailand; sn.nattapon@gmail.com; 3 Department of Helminthology, Faculty of Tropical Medicine, Mahidol University, Bangkok 10400, Thailand; poom.adi@mahidol.ac.th; 4 Department of Social and Environmental Medicine, Faculty of Tropical Medicine, Mahidol University, Bangkok 10400, Thailand; yanin.lim@mahidol.ac.th (Y.L.); phiraphol.chu@mahidol.ac.th (P.C.); yupa.chu@mahidol.ac.th (Y.C.)

**Keywords:** peptide, *Schistosoma mekongi*, biomarker

## Abstract

*Schistosoma mekongi* is found in the lower Mekong river region and causes schistosomiasis. Low sensitivity of diagnosis and development of drug resistance are problems to eliminate this disease. To develop novel therapies and diagnostics for *S. mekongi*, the basic molecular biology of this pathogen needs to be explored. Bioactive peptides have been reported in several worms and play important roles in biological functions. Limited information is available on the *S. mekongi* peptidome. Therefore, this study aimed to identify *S. mekongi* peptides using in silico transcriptome mining and mass spectrometry approaches. Schistosoma peptide components were identified in adult worms, eggs, and infected mouse sera. Thirteen neuropeptide families were identified using in silico predictions from in-house transcriptomic databases of adult *S. mekongi* worms. Using mass spectrometry approaches, 118 peptides (from 54 precursor proteins) and 194 peptides (from 86 precursor proteins) were identified from adult worms and eggs, respectively. Importantly, eight unique peptides of the *S. mekongi* ubiquitin thioesterase, trabid, were identified in infected mouse sera 14, 28, and 56 days after infection. This protein may be a potential target for diagnosis of schistosomiasis. The *S. mekongi* peptide profiles determined in this study could be used for further drug and diagnostic development.

## 1. Introduction

*Schistosoma mekongi* is a causative agent of schistosomiasis in the Mekong region. The Lao People’s Democratic Republic and Cambodia are endemic areas of this disease [1]. In 2017, all countries with endemic schistosomiasis in the Western Pacific Region aimed to achieve interruption of transmission by 2025 and to eliminate transmission by 2030 [2]. The World Health Organization recommends the Kato Katz test as the gold standard for diagnosis of Mekong schistosomiasis. Because this method relies on egg detection in stool using a microscope, it has low sensitivity for mild infections. *S. mekongi* infection generally yields low egg intensity. In some patients, egg shading in feces cannot be observed, and only rectal snipping (a more invasive technique) is able to demonstrate Mekong schistosomiasis [3]. To accomplish the goal of elimination and for effective *S. mekongi* surveillance monitoring, knowledge of the molecular biology of the organism and new interventions for disease control are required.

Bioactive peptides have been reported in many worm species such as *Caenorhabditis elegans* [4] and *Ascaris suum* [5]. These peptides contribute to diverse functions and biological processes. Recently, high-throughput technologies have been developed that allow the discovery of bioactive peptides in pathogenic organisms. In schistosomes, a dermaseptin-like peptide has been identified in acetabular glands of cercariae and has demonstrated antimicrobial, hemolytic, and immunomodulatory properties [6]. A *S. mansoni* neuropeptide—I/Lamide—was identified in *S. mansoni* larval and adult worms. However, no effects of the peptide on aberrant mobility or morphological phenotypes were detected by knockdown experiments [7]. Prohormones involved in reproductive biology were identified using a genome-wide technique in *S. mansoni* and *S. japonicum* [8]. In addition to identification of peptides from parasites, identification of novel markers of *S. mansoni* infection from urine samples has been attempted. Ninety-three percent of infected children could be classified correctly based on their urinary peptide profiles [9]. Hemoglobin-derived peptides were identified in the urine of *S. haematobium*-infected patients even when microhematuria tests were negative [10].

Currently, no information on *S. mekongi* bioactive peptides is available. In this study, the low molecular weight proteins and peptides of *S. mekongi* worm, egg, and infected mouse sera were explored. Because the *S. mekongi* transcriptome is available and was previously published by our group [11], this dataset was used to provide the information of peptides in *S. mekongi* using data mining and proteomic approach. Bioinformatic analyses were applied for assessing the bioactivity of the *S. mekongi* peptidome. The worm and egg peptides are essential for understanding the *S. mekongi* molecular biology, and they also facilitate the schistosome drug development. Moreover, the *S. mekongi* circulating peptides in the infected sera are useful for diagnosis development. This dataset may lead to the development of interventions for the diagnosis and treatment of Mekong schistosomiasis.

## 2. Methods

### 2.1. Identification of Peptidomes from S. mekongi Transcriptome Data

The *S*. *mekongi* adult worm transcriptome dataset [11] was used to identify proteins containing ≤280 amino acids and with molecular weights of ≤30 kDa. The neurofunctions of these proteins were predicted by annotation and neuropeptide domain recognition using the Neuropep database [12]. Non-neuropeptides were further classified by gene ontology using Blast2GO software. The bioactivities of peptides of unknown function were predicted using PeptideRanker software [13]. The significance score threshold was set at 0.8.

### 2.2. Preparation of Worms, Eggs, and Infected Mouse Sera

All animal procedures were approved by the Faculty of Tropical Medicine Animal Care and Use Committee (FTM-ACUC), Mahidol University (approval number FTM-ACUC No. 008/2016). All experiments were performed in accordance with relevant guidelines and regulations. Eight-week-old female ICR mice (25–35 g) were used in this experiment. *S*. *mekongi* cercariae were prepared from *Neotricula aperta* snails without any pre-treatment. ICR mice (N = 6) were anesthetized with Nembutal^®^ (Pentobarbital) 40–60 mg/kg intraperitoneal injection with tuberculin syringe and 26-gauge needle. The mice were exposed with cercariae by abdominal exposure. For a mouse, thirty cercariae were counted under microscope and gently applied to the mouse abdomen by hairpin. Sera were collected pre- and post-infection (days 14, 28, and 56). After 6–8 weeks, infected mice were sacrificed and dissected. Adult worms were flushed out by vascular perfusion using 0.85% sodium chloride. The perfusion solution was transferred to a sedimentation cone. Adult worms settled at the bottom of the cone were collected. Mouse livers and intestines were homogenized in 0.85% sodium chloride. Homogenates were sequentially passed through 80, 120, 160, and 260 mesh stainless steel sieves to separate *S*. *mekongi* eggs from liver and intestinal tissue. The eggs were washed three times with NSS and stored in liquid nitrogen until used.

### 2.3. Peptide Preparation

*S*. *mekongi* adult worms and eggs were dissolved in 8 M urea and sonicated on ice for 10 s. The lysates were centrifuged for 20 min at 20,000 g, 4 °C. The supernatants and mouse sera were individually filtered using Amicon Ultra 0.5 mL centrifugal filters with a molecular weight cutoff of 30 kDa (Millipore, Darmstadt, Germany) by centrifugation for 20 min at 15,000× *g*, 4 °C. The flowthrough peptides were enriched using reverse phase C18 ZipTip chromatography (Millipore). The tips were pre-rinsed with 50% acetonitrile then equilibrated with 0.1% trifluoroacetic acid (TFA). The samples were loaded onto the zips and washed with 0.1% TFA, 80% acetonitrile. The peptides were dried using a speed vacuum (Tomy, Tokyo, Japan).

### 2.4. Mass spectrometry

The peptide solution was resuspended in 0.1% formic acid and then injected on an UltiMate^TM^ 3000 nano-LC system (Dionex, Surrey, UK). The column was an Acclaim PepMap RSLC 75 μm × 15 cm nanoviper C18 with a 2 μm particle size and a 100 Å pore size (Thermo Scientific, Waltham, MA, USA). The LC system was coupled with a MicroToF Q II mass spectrometer (Bruker; Bremen, Germany). Mass ranges were acquired at 500–3500 m/z. MASCOT search engine 2.3 (Matrix Science, Chicago, IL, USA) was used for data analysis. An in-house generated *S*. *mekongi* transcriptomic database was used for searching. The search parameters were as follows: one missed cleavage, trypsin digestion, 0.8 Da peptide tolerance, ±0.8 fragment mass tolerance, acetyl (protein N-terminus), amidated (protein C-terminus), Gln→pyro-Glu (N-terminal Glu), Glu-→pyro-Glu (N-terminal Glu), oxidation (Met), and variable modifications. The significance threshold was 0.05.

### 2.5. Bioinformatic Analysis

The protein sequence of the *S*. *mekongi* ubiquitin thioesterase trabid was retrieved from an in-house transcriptome database. Other ubiquitin thioesterase trabid sequences, including TNN19375.1 (*S*. *japonicum*), XP_018651129.1 (*S*. *mansoni*), XP_012798773.1 (*S*. *haematobium*), TGZ66416.1 (*Opisthorchis felineus*), GAA33082.2 (*Clonorchis sinensis*), OON19543.1 (*Opisthorchis viverrini*), XP_006508032.1 (*M. musculus*), and XP_006717970.1 (*H*. *sapiens*), were retrieved from the nonredundant protein sequence database of the National Center for Biotechnology Information (NCBI). All sequence alignments and calculations of percent identity were performed using Clustal Omega software.

## 3. Results

### 3.1. In Silico Prediction of S. mekongi Peptidomes Using Transcriptome Mining

**Figure 1:**
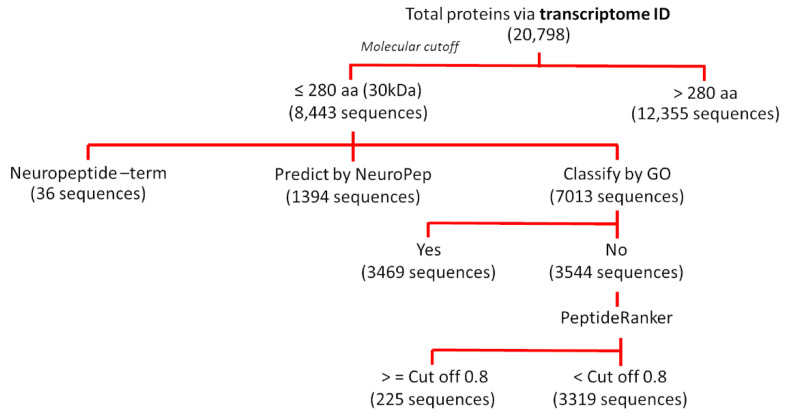
In silico transcriptome mining workflow for *S. mekongi* peptidome prediction.

**Figure 2:**
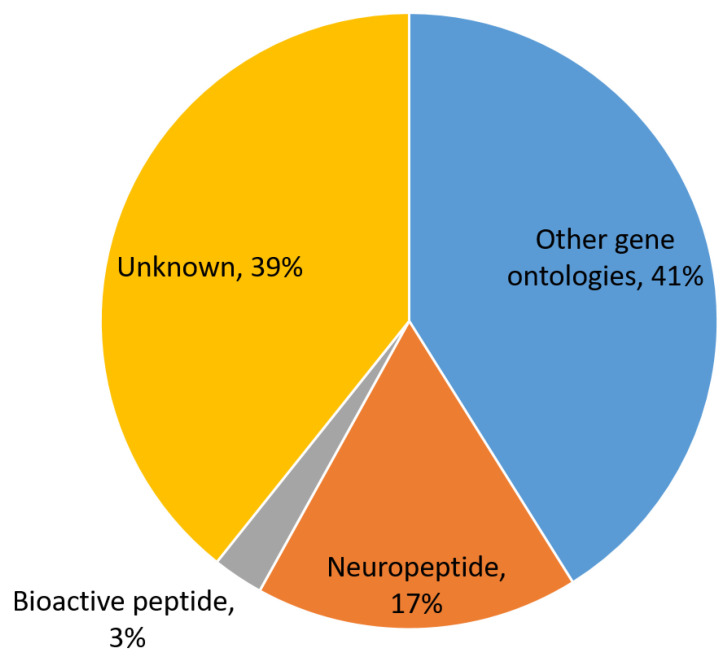
Classification of *S. mekongi* peptidome identified by mining approach.

**Figure 3:**
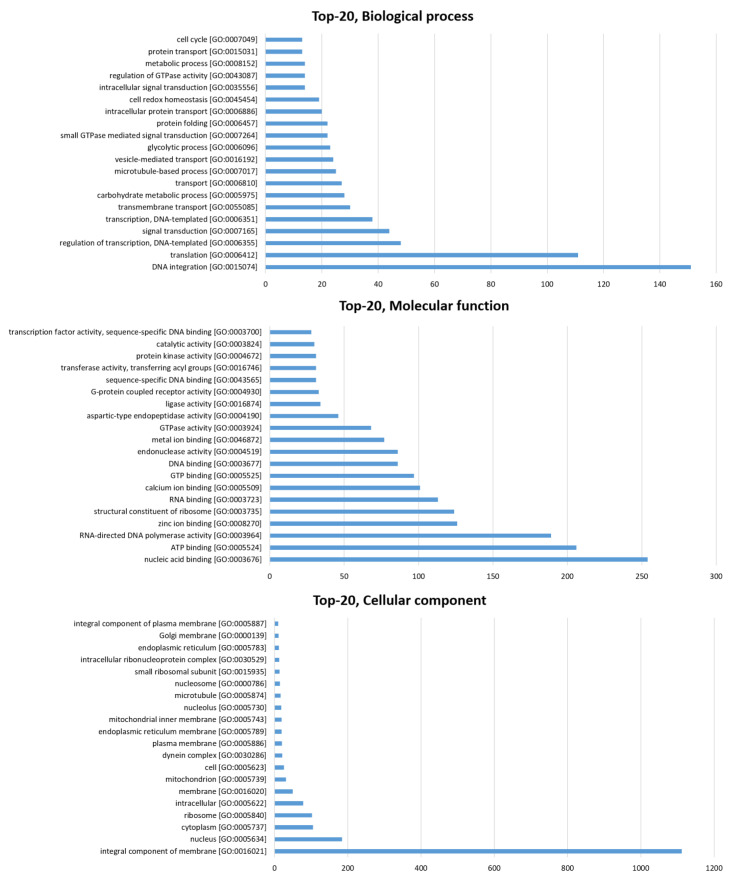
Top-10 most significant gene ontology classification of *S*. *mekongi* peptidome identified by mining approach.

Low molecular weight proteins and peptides with ≤30 kDa molecular weights and ≤280 amino acids in length were retrieved from the *S. mekongi* adult worm transcriptome dataset. A total of 8440 sequences were obtained, and these sequences were further investigated for their biological functions following the workflow in Figure 1. Among them, 17% (1430 sequences), 41% (3469 sequences), 3% (225 sequences), and 39% (3316 sequences) were predicted as neuropeptides, peptides of other gene ontologies, bioactive peptides, and peptides of unknown function, respectively (Figure 2, Supplementary Dataset 1). Neuropeptides could be further classified into 13 families according to a neuropeptide database as shown in Table 1. YGGW-amide related peptide, thyrotropin-releasing hormone (TRH), and vasopressin/oxytocin were the major neuropeptide families of *S. mekongi* adult worms. FMRFamide related peptide, kisspeptin 1 (KiSS1), opioid peptide, LWamide neuropeptide, arthropod hyperglycemic hormone/molt-inhibiting hormone/gonad-inhibiting hormone/vitellogenesis-inhibiting hormone (CHH/MIH/GIH/VIH hormone), serpin, egg-laying hormone (ELH), pyrokinin, neurotensin, and neuropeptide Y family peptides were also observed in *S*. *mekongi*. Non-neuropeptides were further subjected to gene ontology classification using multilevel analysis. A total of 7013 peptides demonstrated gene ontologies. The top 20 classes according to biological process, molecular function, and cellular component terms are shown in Figure 3. DNA integration, translation, and regulation of transcription were the major classes in biological process terms. Nucleic acid binding, ATP binding, and RNA-directed DNA polymerase activity were the main classes in molecular process terms. Integral components of membrane, nucleus, and cytoplasm were the dominant classes in cellular process terms. The bioactivities of peptides whose functions could not be specified by gene ontology were predicted using PeptideRanker. A total of 225 peptides had scores more than 0.8, indicating bioactive properties.

### 3.2. Characterization of S. mekongi Adult Worm and Egg Peptidomes by Mass Spectrometry

**Figure 4:**
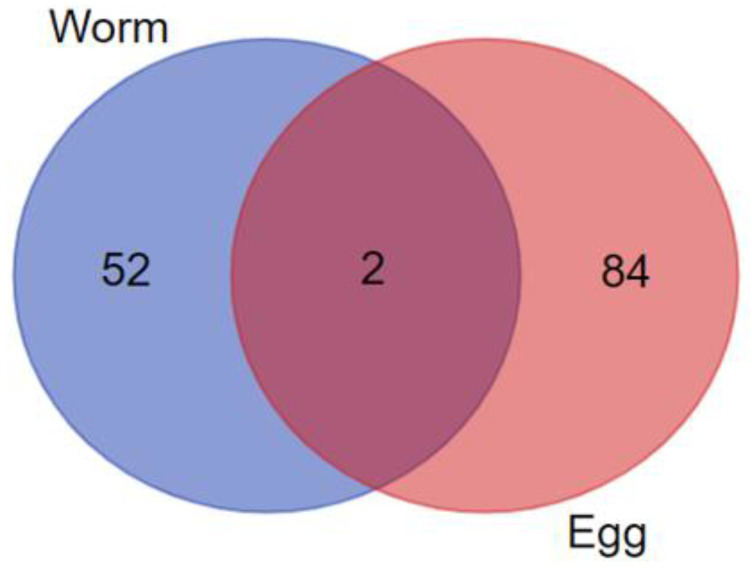
Venn diagram of *S. mekongi* adult worm and egg peptidomes identified by mass spectrometry.

Peptides from *S. mekongi* adult worms and eggs were separated by filtration (30 kDa), purified using stagetip C18 resin, and then analyzed by mass spectrometry. A total of 118 peptides (from 54 precursor proteins) and 194 peptides (from 86 precursor proteins) were identified from adult worms and eggs, respectively (Figure 4 and Supplementary Dataset 2). Only two peptides were observed in both samples: a peptide derived from histone chaperone anti-silencing function protein 1 homolog (DKLDSSNFCENQ) and a peptide derived from an uncharacterized protein (FGQPMMHSGMP). The precursor proteins of these two peptides were not related neuropeptides. The top 10 *S. mekongi* peptides from adult worms and eggs ranked by peptide scores are shown in Supplementary Dataset 3. Peptides NGFSSITTFNVSSSYSKNSNDQDY, KPINAETQFCVSSSVMNNEIFSSL, and SPSQVLFILFMSV demonstrated the highest scores in *S. mekongi* adult worms; their precursor proteins were ethanolamine kinase 1, uncharacterized protein, and AP-3 complex subunit delta-1, respectively. Peptides VNCIESEFL, MHSQHHAKPNVIDKDPDVR, and DNIKKLKTQMEMNMKEQN were the top scoring peptides in *S. mekongi* eggs; their precursor proteins were PDZ domain protein, fatty acid desaturase 1, and an uncharacterized protein, respectively. No neuropeptides were observed in the top 10 ranked peptides from adult worms or eggs. SPSQVLFILFMSV and GFGGHPFSSSG were predicted as bioactive peptides in *S. mekongi* worms and eggs, respectively, using PeptideRanker software with a 0.5 threshold. These peptides may contribute to schistosome molecular function. Further analysis of whole peptides identified by mass spectrometry revealed 17 and 31 proteins identified as precursors of *S. mekongi* neuropeptides in adult worms and eggs, respectively (Supplementary Dataset 4 and 5). The EAAVVSRQHPVKGEC and VSTGGG neuropeptides (QHP and GGG related neuropeptides, respectively) were identified by mass spectrometry from *S. mekongi* adult worms. The QHLSMNPLVESF and QVLMPLGYKVISR neuropeptides (QHL and PLG related neuropeptides, respectively) were identified by mass spectrometry from *S. mekongi* eggs. The amino acid positions of neuropeptide regions within precursor protein sequences were identified using NeuroPep, a neuropeptide software (http://isyslab.info (accessed on 11 April 2021).). Neuropeptides consisting of TKP, PLG and GGG sequences were predominantly found in *S. mekongi* adult worms. Neuropeptides containing GGG, YRI and QHL sequences were mainly observed in *S. mekongi* eggs.

### 3.3. Identification of S. mekongi Peptides in Infected Mouse Sera

**Figure 5:**
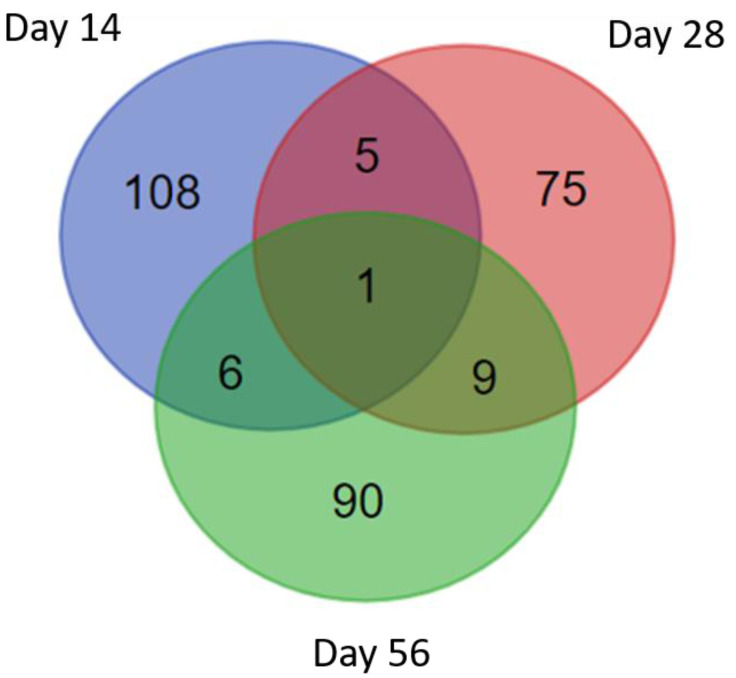
Venn diagram of *S. mekongi* peptides in mouse sera collected pre- and post-infection (days 14, 28 and 56) identified by mass spectrometry.

Peptides were identified from uninfected mouse sera (Day 0) and infected mouse sera (Day 14, Day 28, and Day 56 after infection). Peptide identification was performed by mass spectrometry analysis and by searching against the in-house *S. mekongi* transcriptomic dataset. Any peptides that were identified from uninfected mouse sera were subtracted from those identified from infected mouse sera. A total of 385 peptides (from 120 precursor proteins), 288 peptides (from 90 precursor proteins), and 349 peptides (from 106 precursor proteins) were identified in infected mouse sera 14, 28, and 56 days after infection, respectively (Figure 5). The top 20 *S. mekongi* peptides identified from infected mouse sera are presented in Table 2. The *S. mekongi* peptide profiles in mouse sera 14, 28, and 56 days after infection were dissimilar. These data could be useful for the development of diagnostics to distinguish the early, middle, and late stages of schistosoma infection. Peptides SQFQPHFVVDTMSKGA, QWANLMEKIQASVATNPIITPVAQENQ, and NEVHTMLGQSTEEIRA had the highest scores 14, 28 and 56 days after infection. The precursor protein of the formermost peptide was a cation-transporting ATPase worm, while the precursors of the other two peptides were uncharacterized proteins. *S. mekongi* peptides of the ubiquitin thioesterase trabid (molecular weight 103,015 Da) were consistently observed at 14, 28 and 56 days after infection. The identified peptide sequences of *S. mekongi* ubiquitin thioesterase trabid are presented in Table 3. Eight peptides were specific to *S. mekongi* compared with a *Mus musculus* protein sequence database. The protein sequence of the *S. mekongi* ubiquitin thioesterase trabid was compared with homologs in *S. japonicum, S. mansoni, S. haematobium* and *Homo sapiens*. For other helminths, proteins from *Opisthorchis felineus*, *Clonorchis sinensis* and *Opisthorchis viverrini* which showed the highest percent identity to the *S. mekongi* ubiquitin thioesterase trabid were also used for the comparison. Percent sequence identity is shown in Table 4. The sequence of the ubiquitin thioesterase trabid was conserved among schistosomal species. The percent identities among schistosomal trabid sequences ranged between 81.12% and 93.24%. The sequences of the mouse and human homologs differed substantially. The percent identities between schistosome and human trabid sequences ranged between 39.52% and 40.22%. In addition, percent identities between schistosome and other helminths ranged between 54.18% and 56.23%. Therefore, the schistosomal ubiquitin thioesterase trabid could represent a good candidate for development of diagnostics.

## 4. Discussion

In silico transcriptome mining is a powerful tool for peptidome prediction from biological samples. Using homology-based searches and simple bioinformatics workflows, large peptidomes have recently been predicted for a variety of organisms [14]. However, the mining approach cannot identify peptides produced from post-translational processing. The precursor proteins of processed peptides cannot be identified via the mining technique. Furthermore, the relationships between mRNA and peptide levels are complex and strongly influenced by post-transcriptional and post-translational regulatory mechanisms. Mass spectrometry-based peptidomic approaches are powerful techniques for peptide discovery. This approach can identify peptides derived from proteasome-mediated cleavage of intracellular proteins within the cell [15]. Numerous bioactive peptides requiring proteasome activity for their production are present in tissues and cells [16,17,18]. While the mass spectrometry approach is powerful, peptides at low abundance can be difficult to identify using this approach. Additionally, peptides with extensive post-translational modifications do not effectively ionize, leading to loss of detection [19]. In contrast, in silico transcriptome mining with subsequent bioinformatic peptide prediction is not limited by the above factors. Accordingly, this study used complementary mining and mass spectrometry approaches for *S. mekongi* peptide discovery.

Classical neurotransmitters such as acetylcholine, serotonin and catecholamines have been studied in parasitic platyhelminths and proposed as targets for anthelmintic drugs [20]. However, few studies have addressed the importance of neuropeptides in platyhelminths. In this study, YGGW-amide related peptides were a major *S. mekongi* neuropeptide family identified using a mining approach. Limited information is available on this peptide family in schistosomes. However, this family has been described in *C. elegans.* These peptides are expressed in chemosensory neurons, head neurons, spermatheca, hypoderm, intestine, and embryos of *C. elegans* [21]. Using mass spectrometry approaches, VSTGGG peptides belonging to the YGGW-amide related peptide family were identified in *S. mekongi* adult worms; precursor proteins containing GGG related regions were identified in both *S. mekongi* adult worms and eggs. In *C. elegans*, GGG related peptides are also abundant and are localized in chemosensory neurons, sensory neurons, head neurons, tail neurons, the ventral nerve cord, spermatheca, vulval muscles, and intestine [22].

An important neuropeptide class in flatworms is formed by the FMRFamide-like peptides [23]. Using the in silico transcriptome mining approach, 74 *S. mekongi* FMRFamide-like peptides were identified. Neuropeptides of this family are typically less than 20 amino acids in length and contain an RFamide motif at their C-terminus. FMRFamide-like peptides play a central role in parasite neuromuscular biology. In *S. mansoni*, FMRFamide-like peptides are widespread throughout the nervous system and produce potent myoexcitation [24]. The alteration of levels of *C. elegans* FMRFamide-like peptide signaling impacted locomotory and reproductive behavior. These peptides can bind multiple receptors, making it difficult to indicate the specific receptor being activated by FMRFamide-like peptides [25]. The YIRFamide related peptide is a member of the FMRFamide-like peptide family. It activates muscle contraction by enhancing Ca^2^^+^ influx through sarcolemmal voltage operated Ca^2^^+^ channels (VOCCs). VOCC inhibitors such as nicardipine, verapamil, and methoxyverapamil could inhibit these contractions [26]. YIRFamide related peptides have also been identified in *Dugesia tigrina* [27] and *Bdelloura candida* [28]. In our study, the GYIRF related peptide was detected in *S. mekongi* adult worms but was not observed in eggs. This finding supported the possibility that FMRFamide-like peptides play important roles in *S. mekongi* locomotion. Inhibition of FMRFamide-like peptide receptors might be a practical approach to treat *S. mekongi* infection.

Seven precursor sequences of molluscan ELH were identified in *S. mekongi* using the transcriptome mining approach. ELH is a member of the neuropeptide F family. Peptides in this family are approximately 40 amino acids in length and are characterized by an RxRFamide sequence at the C-terminus. These peptides are orthologs of the neuropeptide Y family in vertebrates. The neuropeptide F and Y families demonstrate high similarity in terms of structure and function [29]. The IRIRFH, DRIRFH and HRIRFN related peptides were detected in *S. mekongi*. In *S. mansoni*, DRIRFH and HRIRFN related peptides were reported. *S. mansoni* ELH potently inhibits Forskolin stimulation of cyclic AMP accumulation, leading to downstream signaling pathway regulation [30]. In *C. elegans*, introduction of a defective mutant ELH into wild-type hermaphrodites or females induced an egg-laying defective phenotype [31]. Additionally, ELH could stimulate ovulation in gastropods, including *Aplysia californica* and *Lymnaea stagnalis* [32]. The *S. mekongi* ELH may also play an important role in regulation of egg-laying.

The protein precursor of GFVRI related peptides (Supplementary Dataset 1: comp7372_seq0, Q5DC41) was identified in *S. mekongi* using the transcriptome mining approach. The GFVRI amide related peptides are a novel family of bioactive helminth neuropeptides that were first reported in *S. mansoni*. Treatment of *S. mansoni* adult worms with this peptide led to significant mobility inhibition [33]. This neuropeptide family might mediate the movement of schistosomal worms.

Both mining and mass spectrometry approaches identified the precursor proteins of PLG related peptides in *S. mekongi* adult worms and eggs. PLG related peptides are part of the vasopressin/oxytocin peptide family. The QVLMPLGYKVISR peptide was identified from *S. mekongi* eggs by mass spectrometry. Vasopressin and oxytocin are neuropeptide hormones. Vasopressinis mainly regulate fluid homeostasis and blood pressure [34]. Oxytocin is involved in uterine contractions and induces milk ejection [35]. Both peptides modulate social behavior, memory, and learning. In *Hirudo medicinalis*, CFIRNCPLG-NH_2_ has been reported as vasopressin/oxytocin peptide family involved in reproductive behavior [7]. This peptide family may be important for *S. mekongi* reproduction.

Kisspeptins are ligands of G-protein coupled receptors. Kisspeptins could initiate signaling associated with secretion of gonadotropin-releasing hormone in humans [36]. A total of 26 KiSS1 precursor sequences were identified from *S. mekongi* adult worms using the transcriptome mining approach. In the sea cucumber *Apostichopus japonicas*, KiSS1 neuropeptides play roles in triggering rapid intracellular mobilization of Ca^2^^+^ and are closely related to seasonal reproduction and metabolism [37]. KiSS1 has been described in teleosts such as the sea bass. The receptors of KiSS1 peptides are mainly expressed in the brain and gonads of sea bass, medaka, and zebrafish. Expression levels of kisspeptins and KiSS receptors in the sea bass testis varied significantly throughout the reproductive cycle [38]. Therefore, KiSS1 neuropeptides may be involved in the reproduction of *S. mekongi*.

Eleven precursor sequences of *S. mekongi* arthropod CHH/MIH/GIH/VIH hormone peptides were detected using the transcriptome mining approach. In crustaceans, the crustacean hyperglycemic hormone (CHH)-family, vitellogenesis-inhibiting hormone (VIH), also known as gonad-inhibiting hormone (GIH), play roles in vitellogenesis [39]. Moreover, MIH is a key endocrine regulator that regulates *Callinectes sapidus* molting and reproduction [40]. The *S. mekongi* arthropod CHH/MIH/GIH/VIH hormone family may have functions in reproduction and development.

The EHP, QHL and QHP related peptides are significant TRH domains and were identified in *S. mekongi* using both transcriptome mining and mass spectrometry approaches. The EAAVVSRQHPVKGEC and QHLSMNPLVESF peptides were identified by mass spectrometry in *S. mekongi* adult worms and eggs, respectively. TRH influences the release of other hormones, including prolactin, growth hormone, vasopressin, insulin, and the neurotransmitters noradrenaline and adrenaline [41]. TRH orthologs were identified in the annelid *Platynereis dumerilii* as well as in *C. elegans* [42]. In *C. elegans,* TRH related peptide is required for growth in body size [43]. Similar to other worms, the TRH family may be involved in *S. mekongi* growth and development.

The precursor sequences of TKP related peptides were dominantly identified in *S. mekongi* adult worms. A total of 178 sequences were identified by transcriptome mining while five and two sequences were detected in *S. mekongi* adult worms and eggs, respectively, by mass spectrometry. The TKP related peptides are part of the N/A neuropeptide family. In humans, these peptides could inhibit macrophage/microglial activation via an unknown mechanism [44]. In mice, TKP related peptides are inhibitors of microglial activation [45]. The role of this peptide in *S. mekongi* adult worms may involve host immune escape through macrophage inhibition.

Although, the peptides identified in this study may influence several behaviors in *S. mekongi*. However, there is a lack of confirmation of the functional information on these peptides. The antioxidant, antimicrobial and other properties of these peptides were not evaluated in this study. Further investigations should be performed to confirm the role of each peptide in *S. mekongi*.

*S. mekongi* infection is currently diagnosed by detection of parasite eggs in stool specimens. It is a low-sensitivity technique, which can underestimate the prevalence and affect mass drug administration. Antibody detection in blood samples is also used to indicate the infection. However, the crude antigen needs to be prepared from the worm or egg which shows variation from batch to batch. Identification of circulating proteins or peptides could improve the diagnosis. The production of monoclonal antibody which binds to these antigens could be one of the strategies for *S. mekongi* diagnosis development. The *S. mekongi* peptide profiles in mouse serum 14, 28, and 56 days after infection were dissimilar. This result is consistent with the life cycle of *S. mekongi*. Approximately 14 days after infection, some *S. mekongi* worms are in the immature stage. They further develop to the mature adult stage and undergo pairing 28 days after infection. Eggs can be observed 56 days after infection. The SQFQPHFVVDTMSKGA, QWANLMEKIQASVATNPIITPVAQENQ, and NEVHTMLGQSTEEIRA peptides had the highest scores 14, 28, and 56 days after infection, respectively. This result could be useful to detect the early, middle, and late stages of *S. mekongi* infection. Eight unique peptides of the *S. mekongi* ubiquitin thioesterase trabid were identified in infected mouse sera starting from 14 days after infection. Comparison of the *S. mekongi* ubiquitin thioesterase trabid protein sequence with homologs from *S. japonicum*, *S. mansoni*, *S. haematobium*, mouse, and human showed that this protein was conserved among schistosoma species. In *Drosophila melanogaster*, the ubiquitin thioesterase trabid is a positive regulator of the Wnt signaling pathway. This pathway regulates numerous processes associated with cell development [46]. In humans, hyperactivation of the Wnt pathway causes colorectal tumors [47]. The *S. mekongi* ubiquitin thioesterase trabid may play roles in growth and development. The protein may be a potential target for early diagnosis of schistosomiasis. However, the individual serum analysis of infected mice needs to be further validated for further diagnosis application.

## 5. Conclusions

Identification of low molecular weight proteins and peptides from *S. mekongi* worm, egg, and infected mouse sera provided the information insight into the molecular biology of schistosome. Besides, the findings could be useful for further schistosomal drug and diagnostic development.

## Figures and Tables

**Table 1:** Family of *S. mekongi* neuropeptides.

Family	Sequences
YGGW-amide related peptide	474
TRH	265
Vasopressin/oxytocin	177
FMRFamide related peptide	74
KISS1	26
Opioid	18
LWamide neuropeptide	14
Arthropod CHH/MIH/GIH/VIH hormone	11
Serpin	10
Molluscan ELH	7
Pyrokinin	3
Neurotensin	1
NPY	1
N/A	491

**Table 2:** Top 20 *S. mekongi* peptides identified from infected mouse serum at 14, 28 and 56 days after infection ranked by peptide scores.

No.	Precursor Protein	Score	E-Value	Peptide	Modification
D14					
1	Putative cation-transporting ATPase worm	56.76	0.00014	SQFQPHFVVDTMSKGA	Oxidation (M)
2	Cyclin-dependent kinase 14	53.61	0.00083	STPLSLVNM	Oxidation (M)
3	Uncharacterized protein	53.46	0.00027	VVYPWTQRYFDSF	
4	Uncharacterized protein	53.35	0.00011	VITSKY	
5	Mitogen-activated protein kinase 15	52.79	0.00025	VITSGYA	
6	Uncharacterized protein	52.22	0.00028	VITSYQ	
7	Uncharacterized protein	52.08	0.00032	VLTSQY	
8	Uncharacterized protein	47.46	0.001	HDGTFISSIGD	
9	Uncharacterized protein	47.23	0.0011	ETSGTSSRVR	Glu- > pyro-Glu (N-term E)
10	SJCHGC05429 protein	44.74	0.0019	VSFITLFM	Oxidation (M)
11	Dynein heavy chain 1 cytosolic	44.03	0.0022	IFNIEPIRAKV	
12	SOSS complex subunit B1	43.75	0.0022	AGDSSSTNR	
13	Vigilin	43.24	0.003	GRGGSKLTELLEGYKRVQV	
14	Uncharacterized protein	42.6	0.0027	LEQENRH	
15	Uncharacterized protein	42.6	0.0027	LEQEEYD	
16	Serine/threonine-protein kinase SIK3	42.45	0.0028	PSIPASNNN	
17	Putative helix-loop-helix zipper protein	41.88	0.0034	SSNTSSNPT	
18	Rho GTPase-activating protein 35	41.81	0.0039	SAFSAPNHS	
19	Helicase	41.29	0.0043	DVGLITGDIKVAPD	
20	Uncharacterized protein	41.24	0.015	SPIKKEEVPAGFSPSEYHLIKKMRDILR	Oxidation (M)
D28					
1	Uncharacterized protein	74.43	2.6E-06	QWANLMEKIQASVATNPIITPVAQENQ	Gln- > pyro-Glu (N-term Q); Oxidation (M)
2	Putative glycosyltransferase	60.49	0.000051	IDVMPSIKTPIE	
3	Putative actin	56.38	0.00013	VFPSIVGRPR	
4	Bifunctional protein NCOAT	52.25	0.00034	NSVAVTLEDL	
5	Putative DNA polymerase delta small subunit	51.8	0.00039	FAGSGQVKPGHSM	
6	Glycosyltransferase 14 family member	50.1	0.00091	TKRQEFF	
7	Uncharacterized protein	45.94	0.0013	THTLTLEN	
8	Coiled-coil domain-containing protein 170	45.9	0.0014	EYVRHNEK	Glu- > pyro-Glu (N-term E)
9	Coiled-coil domain-containing protein 81	45.14	0.0018	EIIFNDIGKLRI	Glu- > pyro-Glu (N-term E)
10	Uncharacterized protein	44.23	0.002	THVDIDKT	
11	Putative multidrug resistance protein 1, 2, 3	44.09	0.0022	QSRANLVTGIIALL	
12	Uncharacterized protein	44.02	0.0044	CLSVMQII	
13	Pogo transposable element with ZNF domain	42.73	0.0036	NIENLDCLECGKCMGD	
14	Putative organic solute transporter	42.32	0.0031	KQATLQFCV	
15	Protein kinase	42.08	0.0037	TEPTIKRMLAENVS	Oxidation (M)
16	Uncharacterized protein	41.47	0.0041	SRQAVQTMGSLFQ	Oxidation (M)
17	Actin bundling/missing in metastasis-related	41.18	0.0045	TTVVSNNGI	
18	Putative Family with sequence similarity 98, member A	40.69	0.0049	GISDRQWS	
19	Nuclear factor 1 C-type	40.67	0.008	LAKENSFF	
20	Uncharacterized protein	40.4	0.0051	SRQAVQTMGSLF	Oxidation (M)
D56					
1	Uncharacterized protein	60.61	0.000054	NEVHTMLGQSTEEIRA	Oxidation (M)
2	Putative organic solute transporter	51.52	0.00037	KQATLQFCV	
3	Uncharacterized protein	49.52	0.00067	LPFLQELDSDQILR	
4	Voltage-dependent calcium channel OS = Schistosoma mansoni GN = Smp_197640 PE = 4 SV = 1	49.14	0.0037	TTSSPLTLIL	
5	Putative importin-beta 2	46.95	0.0015	MLMPPLFEKWNAL	
6	Uncharacterized protein	46.95	0.0011	YDEGKIGIFI	
7	Zinc finger MIZ domain-containing protein 1	46.95	0.0015	PFKCEQPPNGCADAL	
8	Complement component 1 Q subcomponent-binding protein, mitochondrial	45.48	0.0016	EAHPDLRI	Glu- > pyro-Glu (N-term E)
9	39S ribosomal protein L46, mitochondrial	45.03	0.0018	RTRSGVNIFPI	
10	Uncharacterized protein	44.96	0.003	LQLMVPV	
11	Proteasome 26S subunit subunit 4 ATPase	44.88	0.0018	LSFVDKGMLE	Oxidation (M)
12	Uncharacterized protein	44.71	0.002	SVATNPIITPVAQENQ	
13	Teneurin-2	44.14	0.0024	ISILILAFLLAL	
14	Protein kinase	43.75	0.0024	TQCIAYAAGY	
15	Tyrosine-protein kinase Abl	43.57	0.0025	IEAEVALELEKQP	
16	Uncharacterized protein	42.4	0.0056	LEEKMLM	2 Oxidation (M)
17	Protein kinase	42.05	0.0037	TEPTIKRMLAENVS	Oxidation (M)
18	Rhabdoid tumor deletion region protein 1	41.97	0.0047	TTNHGRYTTLNAGAI	
19	Cadherin-related tumor suppressor	41.64	0.0038	MMLSNDLIDS	Oxidation (M)
20	Uncharacterized protein	40.32	0.0048	LTMNTEL	Oxidation (M)

**Table 3:** The identified peptide sequences of *S. mekongi* ubiquitin thioesterase trabid from 14, 28, and 56 days after infection.

Sequence	Score	E-Value	Modification
**Day 14**			
SSNESTADINQTTG *	36.65	0.012	
TSYSPYASPRSSSR *	21.27	4.40 × 10^−1^	
TYTQMPSTNIPLSTPSE *	28.91	0.079	Oxidation (M)
KLSSPLTGNQIHPALQLVFN *	20.94	1.30	
**Day 28**			
HSTLPV	11.45	3.10	
DGGAKWPCGV *	39	0.0073	
SSNESTADINQTTG *	32.69	0.031	
**Day 56**			
DGGAKWPCGV *	34.35	0.021	
VMCFASSPQPLC *	24.27	2.10 × 10^−1^	Oxidation (M)
ESPLTSCGGTTLPV *	26.69	1.20 × 10^−1^	Glu- > pyro-Glu (N-term E)

* Peptide is specific to *S. mekongi.*

**Table 4:** Percent identity matrix of *S. mekongi*, *S. japonicum*, *S. mansoni*, *S. haematobium*, *M. musculus* and *Homo sapiens* ubiquitin thioesterase trabid.

	*S. mekongi*	*S. japonicum*	*S. mansoni*	*S. haematobium*
***S. mekongi***	100	93.24	81.49	81.12
***S. japonicum***	93.24	100	82.02	81.66
***S. mansoni***	81.49	82.02	100	94.86
***S. haematobium***	81.12	81.66	94.86	100
***O. felineus***	55.13	54.77	56.23	56.12
***C. sinensis***	54.89	54.53	55.87	55.65
***O. viverrini***	54.89	54.18	55.87	55.53
***M. musculus***	42.70	42.70	42.25	42.51
***H. sapiens***	39.52	39.84	40.22	40.06

## Supplementary Materials

The following are available online at https://www.mdpi.com/article/10.3390/biom11040559/s1, Table S1: Identification of S. meknogi adult worm peptidome using mining approach, Table S2: Identification of S. mekongi worm, egg and infected serum peptidomes using proteomics, Table S3: Top-10 S. mekongi peptides in adult worm and egg ranked by peptide score, Table S4: The amino acid position of neuropeptide on their precursor protein sequence of S. mekongi adult worm, Table S5: The amino acid position of neuropeptide on their precursor protein sequence of S. mekongi adult egg.
